# Coexistence of Neurofibromatosis Type-1 and MTHFR C677T Gene Mutation in a Young Stroke Patient: A Case Report

**DOI:** 10.1155/2013/735419

**Published:** 2013-02-27

**Authors:** Halim Yilmaz, Gulten Erkin, Haluk Gumus, Lutfiye Nalbant

**Affiliations:** ^1^The Department of Physical Medicine and Rehabilitation, Konya Education and Research Hospital, 42040 Konya, Turkey; ^2^The Department of Neurology, Konya Education and Research Hospital, 42040 Konya, Turkey

## Abstract

In neurofibromatosis type-1 (NF1), cerebrovascular disorders are rarely encountered although vasculopathy is a well-known complication. Several mutations seen in methylenetetrahydrofolate reductase (MTHFR) give rise to the formation of hyperhomocysteinemia and homocystinuria, a considerable risk factor for cardiovascular and cerebrovascular disorders, by leading to enzymatic inactivation. In the paper, a 31-year-old young stroke female patient with the coexistence of neurofibromatosis and MTHFR C677T gene mutation was presented.

## 1. Introduction

The term “stroke” describes findings of focal neurologic syndromes with sudden onset, developing due to vascular events. Strokes experienced under 45 are called young stroke and go definitely undetected in about half of the cases despite large number of investigations [1, 2]. In spite of the course with “café-au-lait” spots and neurofibromas, NF1 may also influence many organs. Although vasculopathy is a known complication in NF1, cerebrovascular disorders are rarely encountered [3, 4]. Seen in MTHFR gene, various mutations cause the formation of hyperhomocysteinemia and homocystinuria, a significant risk factor for cardiovascular and cerebrovascular disorders, by leading to inactivation of enzymes [5]. We reported a 31-year-old woman with young stroke from the coexistence of neurofibromatosis and MTHFR C677T gene mutation.

## 2. Case Report

**Figure 1:**
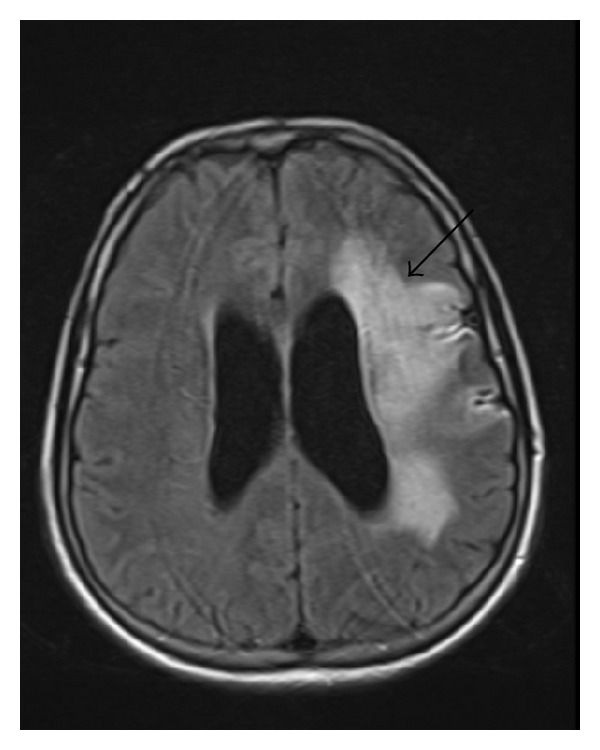
Infarct region compatible with large-size MCA infarct in left frontotemporoparietal lobes on cerebral MRI.

**Figure 2:**
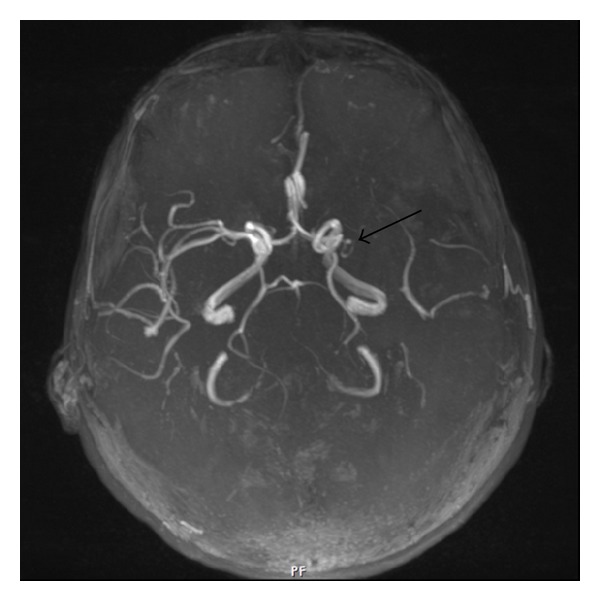
Occlusion from origin of left middle cerebral artery on MRI angiography.

**Figure 3:**
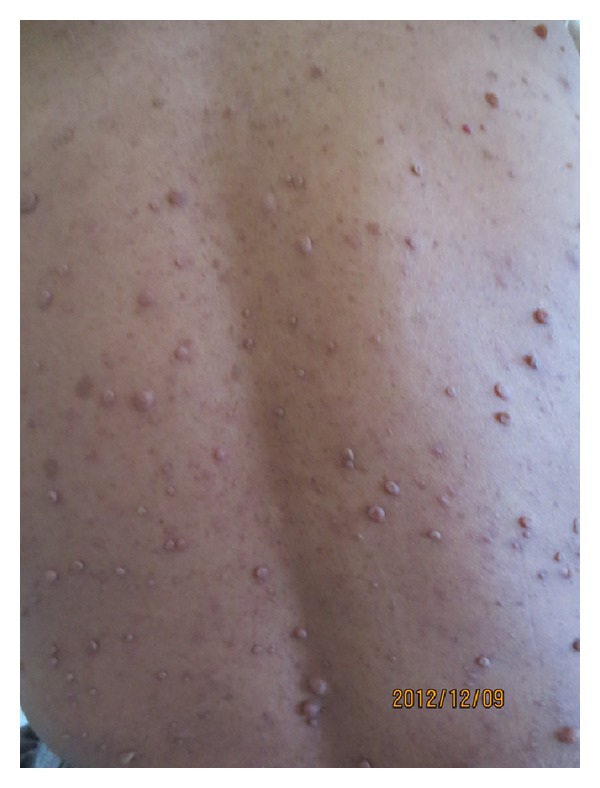
Common neurofibromas and “café-au-lait” spots on thoracolumbar region.

The 31-year-old and 8-year married case was a housewife with a child. In the case applying to a clinic with the complaint of headache nearly 3 months earlier, stroke developed on the same day. No history of systemic disorders such as hypertension, diabetes mellitus and cardiovascular events, drug intake, cigarette smoking and use of alcoholic drinks, spontaneous abortion, and stillbirth was present. Patient's father was determined to have experienced a cerebrovascular event when he was 48. Applying to our clinic with the disability complaints of gait and use of right upper extremity, the patient was admitted with the diagnosis of young stroke. As a result of general and palpable examination on admission, Lisch nodules on both eyes and numerous “café-au-lait” spots and palpable cutaneous neurofibromas which could be seen with naked eyes in lumbar and thoracic regions (Figure 3) were detected. The diagnosis of neurofibromatosis was histopathologically confirmed with biopsy performed with neurofibromas. On neurologic examination, right central facial paralysis, right hemihypoesthesia, and pyramidal findings on right lower and upper extremities were detected. Cranial neurologic examination, except for facial nerves, was within normal limits. Brunstrom's scale was stage II for the right upper extremity, III for the right hand, and V for the right lower extremity. The patient was ambulated for short-distance assisted walking and dependent on routine activities such as feeding, dressing, and gait (functional independence scale score (FIM) = 84). In laboratory tests, whole blood count, lipid levels, electrolytes, tests for liver and kidney functions, coagulation screen, and blood glucose level were within normal limits. Electrocardiography displayed sinus rhythm, and echocardiography and chest X-ray showed no abnormality. In cerebral magnetic resonance imaging (MRI), an infarct region consistent with large-size middle cerebral artery (MCA) infarct extending cortical-subcortical white-matter in left frontotemporoparietal lobes (Figure 1) and, in MRI angiography, an occluded spectrum from the level of left middle cerebral artery origin (Figure 2) were determined. Cranial venous MRI angiography was within normal limits, and scanning showed no abnormality as to vascular occlusive disease. In investigations performed for thrombophilia, heterozygote MTHFR C677T mutation was determined. Homocystein, protein-S, protein-C, antithrombin-III, lipoprotein-a, and factor-5 were within normal limits. Due to the existence of Lisch nodules, “café-au-lait” spots, and cutaneous neurofibromas (Figure 3), NF1 was considered, and with the help of biopsy from cutaneous neurofibromas, the diagnosis of NF1 was supported. Upon investigating the association between NF1 and young stroke cases, a part of patients with NF1 were found to be accompanied by pheochromocytoma, considering that pheochromocytoma may lead to young stroke due to paroxysmal hypertension. Levels of metanephrine and normetanephrine measured in 24 h urine because of pheochromocytoma showed no abnormal results.

As a result, NF1 and heterozygote MTHFR gene mutation were diagnosed in our case with young stroke, and aspirin (300 mg/day) and clopidogrel (75 mg/day) were administered. Due to stroke, the case was started to be rehabilitated in a program (neurophysiologic exercises, neuromuscular electrical stimulation, and gait training). With 4-week intensive rehabilitation program, the case commenced to walk without assistance, and FIM score was 96.

## 3. Discussion

NF1 is an autosomal, dominantly inherited syndrome and encountered about one out of 3000 individuals. NF1 gene is present on chromosome 17 and provides the synthesis of a protein called neurofibromin, functioning as a tumor suppressor [6]. When a mutation is formed in gene on chromosome 17, optic glioma, tumor formations like neurofibromas, pigmented lesions, and typical skeletal lesions may be observed [7]. With the existence of two and more of the criteria by the National Institution of Health, NF1 cases are diagnosed [6, 8]. The case was diagnosed with NF1 due to the determination of numerous neurofibromas and about 20 “café-au-lait” spots in size of 0.5 cm and over in lumbar and thoracic regions and Lisch nodules in both eyes.

The cause of stroke in young adults is different from that in the elderly. Cerebral vascular abnormalities similar to vascular ectasia were reported to be one of the main reasons of young stroke [9]. The prevalence of cerebrovascular dysplasia is 2–5% in patients NF1 [10]. Most of such disorders are composed of occlusion or stenosis in intracranial arteries, aneurysm at a lesser degree, arteriovenous malformations, and moya-moya disease. Our case was also demonstrated to experience left middle cerebral arterial occlusion through MRI. Due to such vascular disorders, cerebral ischemia, hemorrhage and increase of intracranial pressure could be encountered [11], and the mechanism of vascular abnormalities in NF1 still remains unclear. Occurring due to phenotypical expression of NF1 gene, protein of defective neurofibromin is believed to be responsible for the formation of abnormal vascular smooth muscles. The condition results in vascular ectatic alterations and aneurysmal stenosis in blood vessels [12]. Cases related to vascular abnormalities due to NF1 leading to stroke in young adults are present in literature. In a study, Krishnaswami and Vahidassr reported a 43-year-old young stroke patient with pontine infarct due to vascular ectasia associated with NF1 [2]. In another study, a young woman with stroke accompanied by NF1 was reported by Tang et al. [4].

The association between stroke and MTHFR is a known condition. MTHFR plays a key role in the cycle of pholate and contributes to the metabolism of homocystein amino acid [13]. Such clinical features as peripheral neuropathy, growth retardation, hypotonia, stroke, and thrombosis are encountered in severe deficiency of MTHFR where hyperhomocysteinemia and homocystinuria develop [14, 15]. Conditions where MTHFR deficiency is mild are frequently encountered in general population, and the deficiency is suggested to be a risk factor, especially in the development of arterial disorders [14]. It was notably emphasized in a study that an association is present between two common polymorphisms of MTHFR gene (MTHFR C677T and A1298C), and cerebrovascular disorders and venous thrombosis [13]. Also, in a study performed by Morita et al. with 256 stroke patients and 325 controls, a strong association was suggested to be present between MTHFR C677TT genotype and stroke [16]. In our report, left middle cerebral arterial occlusion and mutation of heterozygote MTHFR C677T gene accompanied by NF1 were reported to lead to stroke in a 31-year-old woman patient. Because of giving rise to stroke in young adults, vascular abnormalities stemming from NF1 should meticulously be taken into account in such patients.

Our case may be the first in literature to determine the coexistence of NF1, the cause of young stroke, and accompanying cerebral artery occlusion and mutation of heterozygote MTHFR C677T gene. Therefore, although the association between stroke and NF1 is rarely encountered in daily practice in young patients applying to clinics with the complaint of stroke, diagnosis of NF1 should be taken into account, and the existence of NF1 should be investigated via a careful dermatologic examination. In addition, in young patients with the complaint of stroke, MTHFR C677T gene mutation should be considered as etiologic reason, and the mutation should be kept in mind to be accompanied by NF1. Young patients diagnosed with NF1 may be suggested to be closely followed up as to neurologic symptoms and complications, and stroke risks in future should be evaluated via cerebral vascular imaging in such patients.
